# New Copolymers of Vinylphosphonic Acid with Hydrophilic Monomers and Their Eu^3+^ Complexes

**DOI:** 10.3390/polym14030590

**Published:** 2022-01-31

**Authors:** Olga Nazarova, Elena Chesnokova, Tatyana Nekrasova, Yulia Zolotova, Anatoliy Dobrodumov, Elena Vlasova, Andrei Fischer, Marina Bezrukova, Eugeniy Panarin

**Affiliations:** Federal State Budgetary Institution of Science Institute of Macromolecular Compounds, Russian Academy of Sciences (IMC RAS), V.O. Bolshoy pr. 31, 199004 Saint Petersburg, Russia; lenavitalena@mail.ru (E.C.); polar@macro.ru (T.N.); julia.i.zolotova@gmail.com (Y.Z.); anatoliy.dob@gmail.com (A.D.); spectra@imc.macro.ru (E.V.); andreasfischer@mail.ru (A.F.); bezrukova@imc.macro.ru (M.B.); lab.2@mail.ru (E.P.)

**Keywords:** water-soluble copolymers of vinylphosphonic acid, 2-deoxy-2-methacrylamido-D-glucose, 4-acryloylmorpholine, complexes of Eu^3+^

## Abstract

Free radical copolymerization is used for the synthesis of novel water-soluble copolymers of vinylphosphonic acid with 2-deoxy-2-methacrylamido-D-glucose or 4-acryloylmorpholine, with varied compositions and molecular masses, as well as for the synthesis of copolymers of vinylphosphonic acid with acrylamide. The obtained copolymers contain 6–97 mol.% of vinylphosphonic acid units, and their molecular masses vary from 5 × 10^3^ to 310 × 10^3^. The monomer reactivity ratios of vinylphosphonic acid and 2-deoxy-2-methacrylamido-D-glucose in copolymerization are determined for the first time, and their values are 0.04 and 9.02, correspondingly. It is demonstrated that the synthesized copolymers form luminescent mixed-ligand complexes with Eu^3+^, thenoyltrifluoroacetone, and phenanthroline. The influence of the comonomer’s nature on the intensity of the luminescence of complex solutions is revealed.

## 1. Introduction

Poly(vinylphosphonic acid) (PVPA) is applied as a component of anticorrosive coatings, polymeric electrolyte membranes, dental cements, and in bone tissue engineering [1,2,3,4]. A promising direction for the use of VPA polymers is associated with their antimicrobial and antiviral activities; in particular, they are active against coronavirus infection [5,6].

It is also known that VPA polymers are capable of binding with various metal cations [7] and luminescent metal-polymer complexes, for example, with Eu^3+^. Based on this, VPA polymers can potentially be used to visualize the interaction of polymers with viral particles and cells.

Currently, various fluorophores are used for such visualization, including fluorescein, dansyl chloride, and rhodamine [8]. The coordination of compounds with Eu^3+^ has attracted considerable interest because of their fascinating luminescence properties, such as the quasi-monochromaticity of their radiation (the half-width of the luminescence bands is 5–10 nm, while for organic chromophores, it is more than 100 nm), the stability of their radiation over time, the independence of the position of the luminescence bands from the nature of the ligand and solvent, a large Stokes shift in complexes, and the high values of excited state lifetimes (≈1000 µs), meaning that they may be used as luminescent sensors for visualization in medicine and biology [9]. It is known that the use of polymer complexes in biology and medicine makes it possible to prolong their action in comparison with low molecular weight complexes [10]. Due to the high binding constants of VPA with ions of polyvalent ions (lanthanides) [11], the preparation of Eu^3+^ complexes with such polymers is of considerable interest. However, these complexes do not luminesce. In this connection, it is relevant to study the possibility of obtaining luminescent complexes using the “antenna effect” [12], based on the transfer of electronic excitation energy from the triplet level of the ligand to the excited level of the lanthanide, where luminescence occurs. It should be taken into account that the efficiency of energy transfer depends not only on the energy of the triplet level of the ligand, but also on the nature of the substituents in it, their interaction with the environment, and the concentration of the complexing units in the macromolecule [13,14].

One way to vary the properties is to use copolymers. The use of comonomers of different natures makes it possible to change the microenvironment of the chelate group.

**Figure 1:**
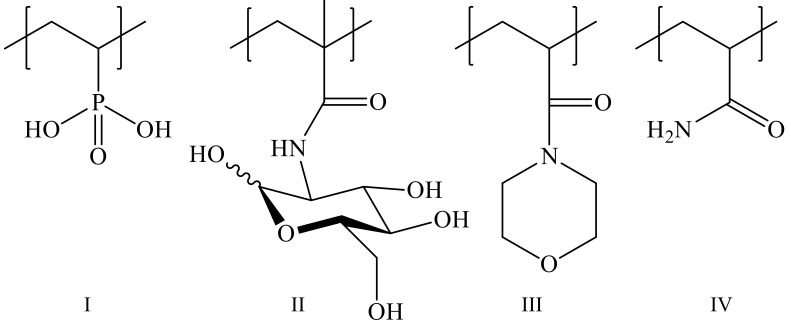
VPA (**I**), MAG (**II**), 4-AM (**III**), AA (**IV**) units.

The aim of this work was to develop synthetic methods to prepare and study novel original copolymers of VPA with 2-deoxy-2-methacrylamido-D-glucose (MAG) and 4-acryloylmorpholine (4-AM), with varied compositions and molecular masses; to prepare VPA copolymers with acrylamide (AA) (Figure 1); and to estimate the ability of the synthesized copolymers to form luminescent complexes with Eu^3+^ ions, and examine the photophysical properties of such complexes.

The random double and triple copolymers of VPA with methyl acrylate, acrylonitrile [15], acrylic and methacrylic acids [4,16], acrylamide [17], styrene [18], N-vinylpyrrolidone [19], N-vinylimidazole [20], etc., are known. The graft and block copolymers of VPA have been synthesized [2,3,21]. However, the VPA copolymers with MAG and 4-AM and their luminescent complexes with Eu^3+^ are not described in the literature.

## 2. Materials and Methods

### 2.1. Materials

VPA, 4-AM, 2,2-dimethylformamide (DMFA), 2,2′-azobisisobutyronitrile (AIBN), and 2,2′-azobis(2-methylpropionamidine) dihydrochloride (AMP) were purchased from Aldrich (Germany). VPA was washed with diethyl ether to remove the inhibitor and dried under vacuum. Then, 4-AM and DMFA were distilled under vacuum. MAG was synthesized according to the technique described in [22]. The physico-chemical parameters of all substances corresponded to the literature values.

### 2.2. Methods

#### 2.2.1. Synthesis of (Co)Polymers

The VPA homopolymer and copolymers were synthesized via free radical copolymerization in an argon atmosphere at 60 °C for 24 h. When the process was carried out in organic solvents, AIBN was used as an initiator; in aqueous solutions, AMP was employed. In the experiments for the determination of monomer reactivity ratios, the copolymer yields did not exceed 5 wt.%.

To purify the copolymers from low molecular weight admixtures, they were dialyzed against water. Spectra/Por 7 membranes (Spectrum Laboratories, Inc., Seguin, TX, USA) were used; these membranes allow one to remove substances with molecular masses below 1000. Then, the products were isolated by freeze drying.

#### 2.2.2. Compositions of Copolymers

The compositions of the copolymers were determined using 1H and 31P NMR spectroscopy methods. The NMR spectra of the solutions of samples in D_2_O were registered with the aid of a Bruker Avance 400 spectrometer (Bruker, Karlsruhe, Germany). In addition, 2-Methacryloyloxyethyl phosphorylcholine (MPC) was used as an external standard.

#### 2.2.3. IR Spectroscopy

The IR spectra of samples were obtained at room temperature in the 400–4000 cm^−1^ wavenumber range (resolution: 4 cm^−1^; number of scans: 30) using a “Vertex 70” FTIR spectrometer (Bruker, Ettlingen, Germany) equipped with a ZnSe attenuated total reflection (ATR) attachment (“Pike Technologies”, Madison, WI, USA). During the registration of the ATR spectra, a correction was made that took into account light penetration depth as a function of wavelength.

#### 2.2.4. Determination of Molecular Masses of Polymers

Diffusion coefficients *D* and sedimentation coefficients *s* were measured using the experimental setups equipped with polarization interferometers described in [23]. The polarization interferometers installed in the ultracentrifuge and the diffusiometer made it possible to carry out measurements at relatively low concentrations of samples (less than 0.15 × 10^−2^ g/cm^3^); besides, it was not necessary to study concentration dependences.

The molecular masses (MM) of the samples were calculated from the values of diffusion coefficients *D* and sedimentation coefficients *s*, measured in 0.1 N NaCl solution at 24 °C according to the Svedberg formula:

(1) $$M_{sD}=\left(s/D\right)\times RT/\left(1-\bar{v}\rho_{0}\right)$$

where *R* is the universal gas constant, *T* is the absolute temperature (K), $\bar{v}$ is the partial specific volume determined by pycnometry, and *ρ_o_* is the density of a solvent [23]. The $\bar{v}$ values of the copolymers were calculated additively from the mass densities ($\bar{v}$^−1^) of the copolymer components, taking into account sample compositions. The values measured by pycnometry were equal to 0.529 cm^3^/g for poly(vinylphosphonic acid), 0.667 cm^3^/g for poly-MAG, 0.74 cm^3^/g for poly(4-acryloylmorpholine), and 0.77 cm^3^/g for polyacrylamide. The values of the buoyancy factor (1 − *νρ*_0_) ranged from 0.24 to 0.46, depending on the copolymer composition.

#### 2.2.5. Synthesis of Complexes with Eu^3+^

Thenoyltrifluoroacetone (TTA) and phenanthroline (PHEN) were used as sensitizers for Eu^3+^ luminescence. Calculated amounts of solutions of EuCl_3_⋅6H_2_O, TTA, and PHEN (concentration: 5 × 10^−3^ mol/L) were consequently added to the aqueous solution of a copolymer (concentration of VPA units: 1 × 10^−4^ mol/L). The following molar ratios were used: VPA unit/Eu^3+^/TTA/PNEN = 3:1:2:1. The solutions were left to stand for 1 h at room temperature.

#### 2.2.6. UV-Vis Spectra

The electronic absorption spectra of copolymer solutions were obtained with the use of an SF-256 UVI spectrophotometer (“OOO LOMO Photonika”, Russia).

#### 2.2.7. Excitation and Luminescence Spectra

The excitation and luminescence spectra of solutions were registered with the use of an LS-100 spectrofluorometer (“PTI”, London, ON, Canada). The widths of the entrance and exit slits of the monochromator were equal to 4 nm. The measurements were carried out at 25 °C in a 1 cm quartz cuvette.

## 3. Results and Discussion

Table 1 presents the synthesis conditions and characteristics of the prepared MPC copolymers.

**Figure 2:**
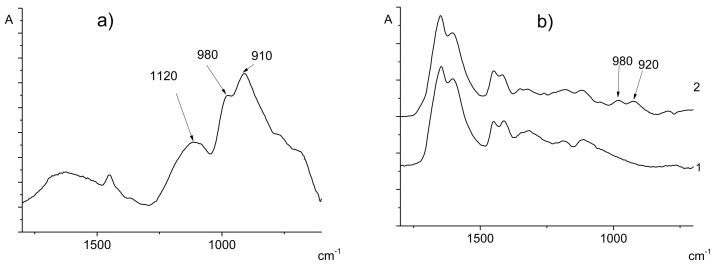
FTIR spectra of VPA homopolymer (**a**), AA homopolymer (**b**,1), AA-VPA copolymer (Experiment 11, Table 1) (**b**,2).

Figure 2 demonstrates FTIR spectra of the synthesized PVA-AA copolymer and the corresponding homopolymers. The spectrum of the VPA homopolymer contains the following bands: 1120 cm^−1^ (hydrogen bonded P = O P = O participating; 980 cm^−1^ and 910 cm^−1^ (P-O vibrations) [24,25]. The spectrum of the AA homopolymer includes the bands near 1650 cm^−1^ that correspond to the C = O vibration of the amide I band. The spectrum of the AA-VPA copolymer contains all the indicated bands of both comonomer units (Figure 2).

**Figure 3:**
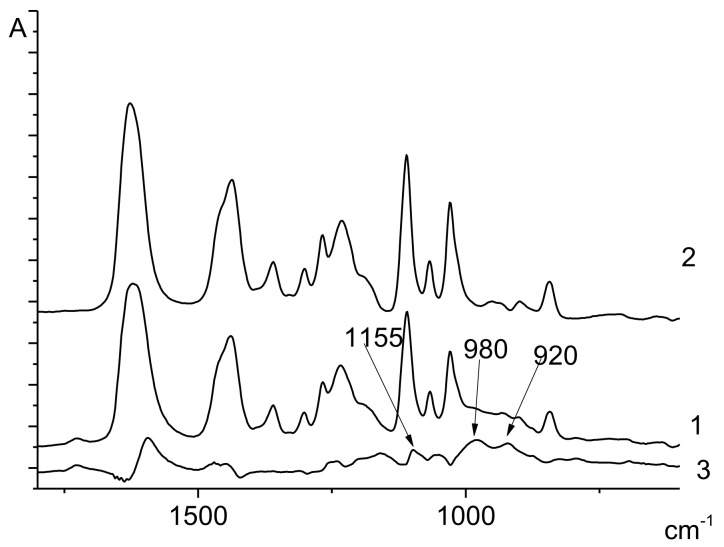
FTIR spectra of poly(4-acryloylmorpholine) (1), 4-AM-VPA copolymer (Experiment 5, Table 1) (2), the difference spectrum (3).

The spectrum of poly(4-acryloylmorpholine) contains the characteristic bands at 1620 cm^−1^ (amide I) and 1113 cm^−1^ (related to vibrations of the C-O-C fragment of the cyclic ether). Unlike the bands of the 4-AM units, the bands related to the VPA units in the spectrum of the 4-AM-VPA copolymer are poorly resolved; for the purpose of the detailed study of these peaks, the difference spectrum of the 4-AM-VPA copolymer and poly(4-acryloylmorpholine) was obtained. In this spectrum, the peaks at 1155 cm^−1^, 980 cm^−1^, and 920 cm^−1^, typical for the VPA units, were observed (Figure 3).

**Figure 4:**
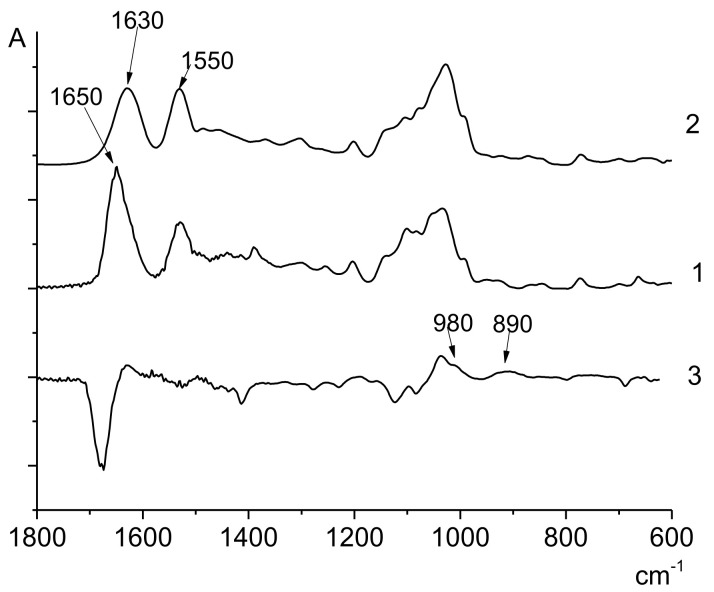
FTIR spectra of MAG homopolymer (**1**), MAG-VPA copolymer (**2**) (Experiment 3, Table 1), the difference spectrum (**3**).

The spectrum of the MAG homopolymer (Figure 4) contains the characteristic amide I (1650 cm^−1^) and amide II (1550 cm^−1^) bands, as well as the peak related to the vibrations of the glycoside ring (1000 cm^−1^). The amide I band of the MAG units in the spectrum of the MAG-VPA copolymer becomes less intense, and it is widened and shifted (from 1650 cm^−1^ to 1630 cm^−1^); besides, the shape of the band attributed to the glycoside ring near 1000 cm^−1^ changes. The peaks attributed to the VPA units are poorly resolved. As in the case of the 4-AM-VPA copolymer, the difference spectrum (obtained from MAG-VPA and the MAG homopolymer) contains the bands at 980 cm^−1^ and 890 cm^−1^ typical of VPA units. The shift of one band from 910 cm^−1^ to 890 cm^−1^, and the changes in the MAG bands, are apparently related to the differences in the microenvironments of these units in the homopolymer or the copolymer.

**Figure 5:**
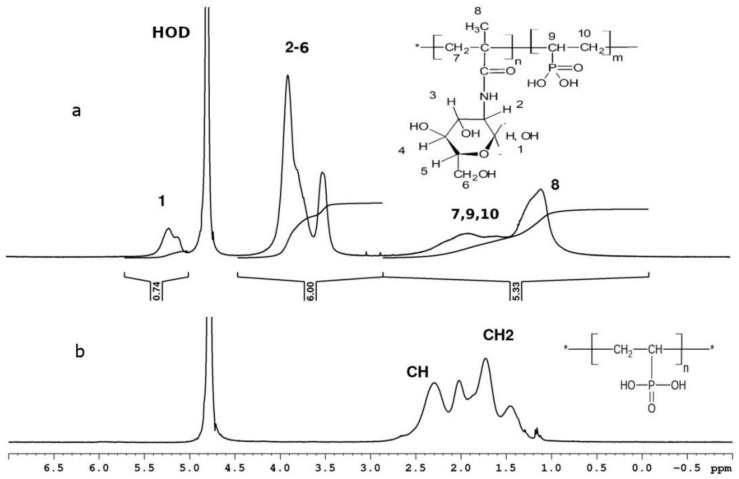
^1^H NMR spectra of MAG-VPA copolymer (Experiment 3, Table 1) (**a**), PVPA (**b**) in D_2_O.

**Figure 6:**
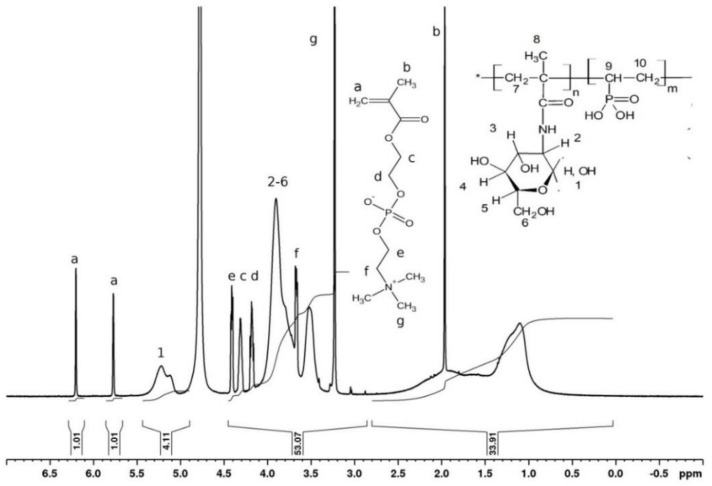
^1^H NMR spectrum of the mixture containing MAG–VPA copolymer (Experiment 3, Table 1) and MPC in D_2_O.

The compositions of the copolymers were determined through NMR spectroscopy [4]. Unfortunately, in all cases in the ^1^H NMR spectra of all copolymers, the signals from the protons of the VPA backbone at around 1.0–2.5 ppm (Figure 5 and Figure 6) overlap with the proton signals of the comonomers. Therefore, it is impossible to determine explicitly the composition of the copolymers from the ^1^H NMR spectra only; for this reason, a combination of 1H and ^31^P NMR spectroscopy was used. A phosphorus-containing compound (2-methacryloyloxyethyl phosphorylcholine (MPC)) was used as a concentration reference mark, because in all cases, the signals of the two protons from the MPC of the double bond (that are located near 5.5–6.5 ppm, Figure 6) are not overlapped with the signals of the comonomer units.

For example, in the ^1^H NMR spectrum of the mixture of copolymer VPA-MAG (Experiment 3, Table 1) and MPC, both signals of protons 2–6 of the MAG ring (six protons) and the signals of 17 protons c, d, e, f, and g of MPC are observed in the 3.0–4.5 ppm range (Figure 6). When the intensities of the MPC signals in the 5.5–6.5 ppm range were normalized to unity, the integral intensity of the signals of protons 2–6 of the MAG ring (A) was equal to the integral intensity of all signals in the 3.0–4.5 ppm range (B), minus the intensity of the 17 MPC protons. Correspondingly, the integral intensity per one MAG proton (C) is equal to C = A/6 = (B − 17)/6. The molar ratio between MAG and MPC is MAG:MPC = C:1.

**Figure 7:**
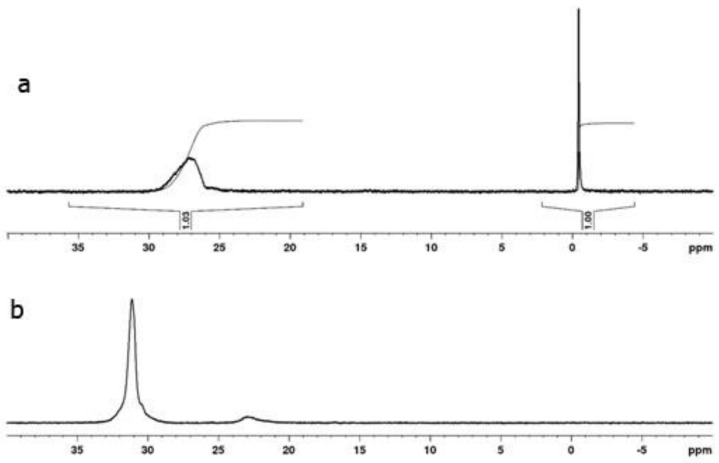
^31^P NMR spectra of the mixture of MAG–VPA copolymer with MPC (**a**) and PVPA (**b**) in D_2_O.

The ^31^P NMR spectrum presented in Figure 7 was used to determine the molar ratio Y = VPA/MPC. It was calculated as a ratio of the integral intensities of the MPC signal near −0.7 ppm (D) and the copolymer signals in the 20–35 ppm area (E) (Y = E/D) (see Figure 7). Then, the molar ratio between the copolymer units in a sample can be calculated as F = C:Y = C:E/D. For copolymer 3 (Table 1), F = (53.07 − 17)/6:1.01 = 6:1. Therefore, the molar composition is the following: 86 mol.% of MAG and 14 mol.% of VPA.

The compositions of all the synthesized copolymers were calculated in a similar way.

In all cases, the copolymers contained less VPA units than the initial reaction mixture (Table 1), which is especially noticeable in the case of the MAG-containing copolymers.

Using water instead of organic solvents (DMFA or methanol), other things being the same, leads to an increase in the molecular masses of the products (Experiments 5, 6, and 9–11 in Table 1). This is due to the extremely low values of the chain transfer constants in water [26,27].

All copolymers were soluble in water.

The described copolymers MAG-VPF and 4-AM-VPA were obtained for the first time; thus, there are no literature data on the reactivities of the used comonomers in the course of copolymerization. It was interesting to estimate their relative activities. The compositions of the MAG-VPA copolymers were determined when the yields did not exceed 5 wt.%. It was found that at all comonomer ratios, the copolymers formed at the early stages of the copolymerization process were heavily depleted with VPA units (in comparison with the initial monomer mixture).

The relative reactivities of the monomers were calculated using the Fineman–Ross, and the Kelen–Tüdös methods [28,29]. The following values were obtained: r_VPA_ = 0.04; r_MAG_ = 9.02. Thus, VPA is a considerably less active comonomer during copolymerization with MAG. The low activity of VPA has been reported earlier in copolymerization with acrylic acid [4] and N-vinylpyrrolidone [19]. According to the suggested mechanism, VPA polymerization proceeds with the formation of VPA anhydride cycles and the formation of various types of radicals. It is assumed that this process leads to a decrease in the relative reactivity of VPA during polymerization [4,30].

In all cases, an increase in the content of this monomer, with low reactivity in the initial comonomer mixture, led to a decrease in copolymer yield and the M_SD_ of the product (Table 1). This effect was registered in work [16].

**Figure 8:**
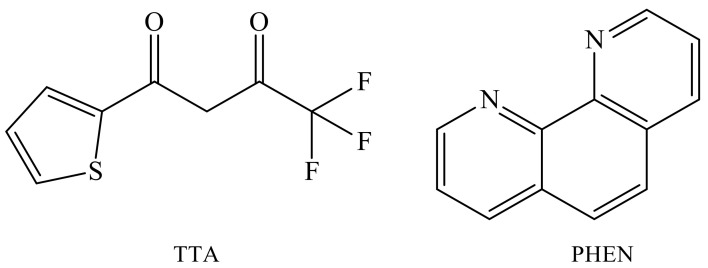
TTA and PHEN structures.

The binding constants of lanthanide ions by phosphonic acid and its derivatives (Kb) are in the range of 10^5^–10^6^ (17,600–313,000) [11]; however, the forming complexes do not exhibit luminesce. This is due to the fact that the luminescence intensity of individual lanthanide ions is due to f–f transitions within individual ions, since these transitions are forbidden (the Laporte rule). Therefore, the absorption and, accordingly, the luminescence of lanthanide ions are extremely low. The discovery of the luminescence sensitization of lanthanide ions by an organic ligand, the “antenna effect” [12], allows one to bypass the direct excitation of the lanthanide ion, which is prohibited by the selection rules. The introduction of an organic ligand that effectively absorbs the energy of electron excitation and transfers it from the triplet level to the resonance level of the lanthanide, from which luminescence occurs, is widely used in the formation of the luminescence complexes of lanthanide ions. The position of the triplet level of the ligand in relation to the resonant level of the lanthanide ion [31] is the basic factor for improving the efficiency of sensitization. In phosphonic acid (co)polymers, the lanthanide ion can coordinate two phosphonate groups. However, the OH groups in phosphonates are unable to saturate the coordination sphere of lanthanide ions. Owing to the high coordination numbers (8–10) of the lanthanide complexes [14], it is possible to introduce a neutral ligand and vary the luminescent properties in a regulable manner. In this work, thenoyltrifluoroacetone (TTA) and phenanthroline (PHEN) molecules were used as luminescence sensitizers for Eu^3+^ ions (Figure 8).

**Figure 9:**
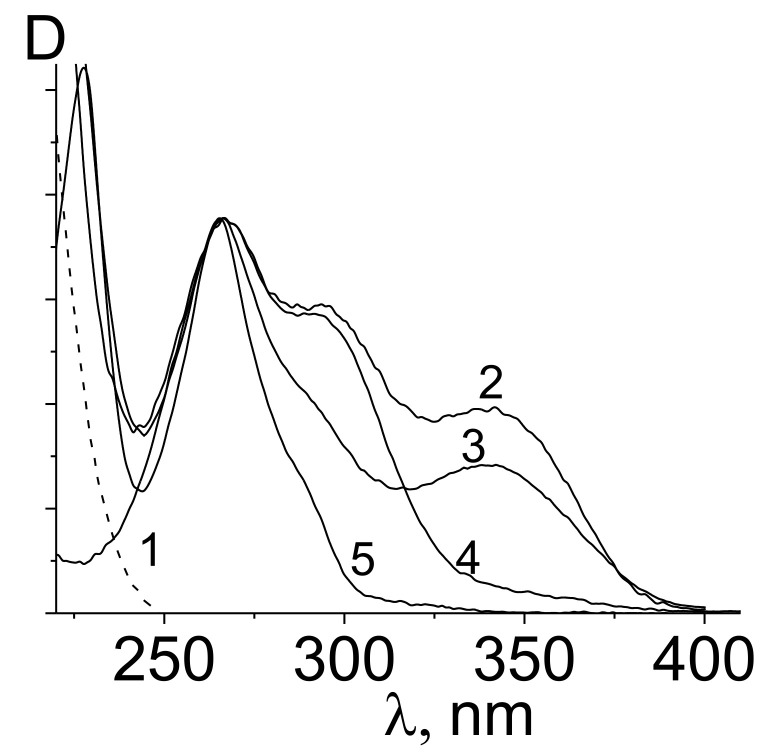
Normalized electronic absorption spectra of aqueous solutions: Eu^3+^/4-AM-VPA copolymer (Experiment 5, Table 1) (**1**), Eu^3+^/4-AM-VPA copolymer/TTA (**2**), Eu^3+^/4-AM-VPA copolymer/TTA/PHEN (**3**), TTA (**4**), and phenanthroline (**5**).

Figure 9 presents as an example of a normalized electronic absorption spectra pacтвopoв of the following solutions: Eu^3+^/4-AM-VPA copolymer (Experiment 5, Table 1) (1), Eu^3+^/4-AM-VPA copolymer/TTA (2), Eu^3+^/4-AM-VPA copolymer/TTA/PHEN (3), TTA (4), and phenanthroline (5). The UV spectra were normalized to the optical density of the TTA solution in the band at 266 nm.

The UV spectra of the solutions of the VPA-MAG and VPA-AA copolymers have a similar shape.

It is known that TTA forms complexes with multivalent metal ions, including lanthanides (which is indicated by the appearance of a new band in the long-wavelength region of the TTA electronic spectra). Spectra (2) and (3) contain the peak at λ = 340 nm that indicates the formation of the complex with Eu^3+^.

**Figure 10:**
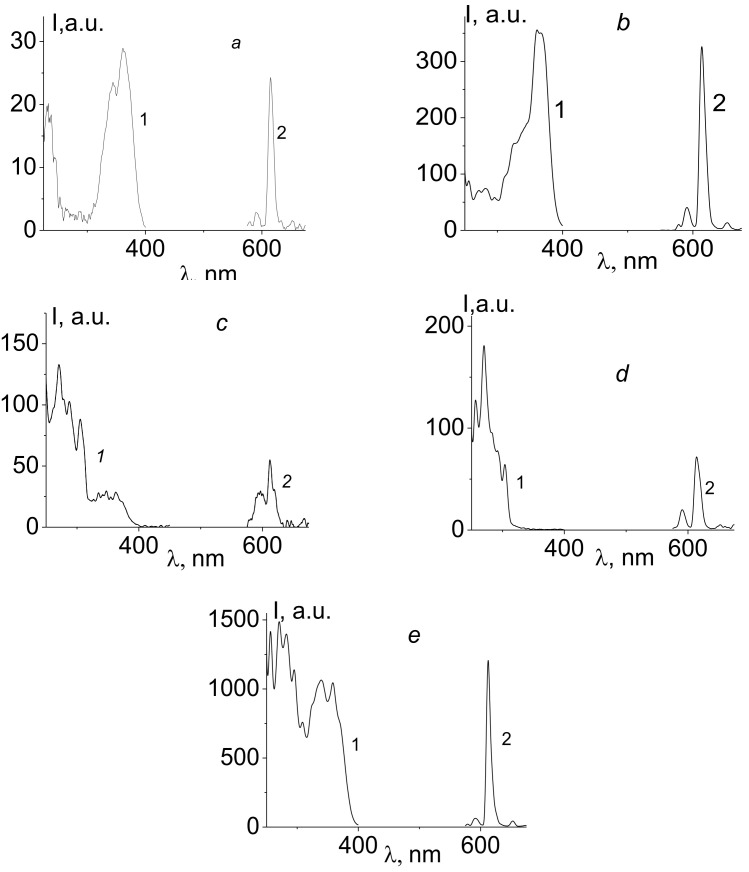
Excitation (1) and photoluminescence (2) spectra of solutions of Eu^3+^/TTA (**a**), Eu^3+^/4-AM-VPA/TTA (**b**), Eu^3+^/PHEN (**c**), Eu^3+^/4-AM-VPA/PHEN (**d**), and Eu^3+^/4-AM-VPA/TTA/PHEN (**e**).

Figure 10 shows the excitation (1) and photoluminescence (2) spectra of Eu^3+^/TTA (a), solutions of Eu^3+^/4-AM-VPA/TTA (b), solutions of Eu^3+^/4-AM-VPA/PHEN (c), solutions of Eu^3+^/PHEN (d), and solutions of Eu^3+^/4-AM-VPA/TTA/Phen (e).

It was found that the VPA copolymers with MAG, 4-AM, and AA form luminescent complexes with Eu^3+^ in the presence of TTA or PHEN. With Eu^3+^ in a solution of a polymer complex, the so-called “polymer effect” is due to the displacement of water molecules from the coordination sphere of a low molecular complex and the substitution of a part of the water molecules by copolymer phosphonate groups.

The comparison of the absorption and excitation spectra of the complex Eu^3+^/4-AM-VPA copolymer/TTA (Figure 9, spectrum 2 и Figure 10b, spectrum 1) shows that their shapes differ. Pronounced differences in the shape between the absorption and excitation spectra in the lanthanide complexes indicate the sensitization efficiency, because the ratio between the bands, which originated from the ligand absorption and from the absorption of the lanthanide ion itself, depends on both the molar extinction coefficient and also on the sensitization [13]. Since the photoluminescence spectra were obtained at different voltages on the photomultiplier, the normalized *I*_614_ values related to *I*_614_ of the Eu^3+^/TTA complex, which is shown in Table 2.

It has already been noted that the Eu^3+^/VPA polymer and the Eu^3+^/4-AM-VPA copolymer complexes do not exhibit significant luminescence, since the direct excitation of the Eu^3+^lanthanide ion is impossible. When TTA is added to a solution of Eu^3+^/4-AM-VPA, intense luminescence appears in the solution (Figure 10b, spectrum 2). The spectrum contains bands characteristic of Eu^3+^, with λmax = 580, 595, 614, 655, and 702 nm, which is related to transitions from the ^5^D_0_ state to the ^7^F _j_(1–5) Eu^3+^ levels, respectively.

Table 2 shows that luminescence of Eu^3+^ ions in the copolymer solution containing TTA or PHEN is enhanced by more than an order of magnitude as compared with that of the Eu^3+^/TTA in an aqueous solution at the same concentrations. The weak luminescence of the Eu^3+^/TTA solution is due to the low value of the formation constant of the Eu^3+^/TTA complex in aqueous solutions (without a polymer), where logK= −3.4 [32]. A similar effect is observed for the complex Eu^3+^/4-AM-VPA copolymer/PHEN (Figure 10c, spectrum 2).

The enhanced luminescence of Eu^3+^/TTA or Eu^3+^/PHEN in a solution of a polymer complex, the so-called “polymer effect”, is due to the displacement of water molecules from the coordination sphere of a low molecular complex and the substitution of a part of the water molecules by copolymer phosphonate groups.

In a number of publications [33,34,35], it has been demonstrated that an increase in europium luminescence intensity may be caused by the formation of mixed-ligand complexes. As it is seen in Figure 10, introducing PHEN to the solution of Eu^3+^/4-AM-VPA copolymer/TTA (in the ratio [TTA]/[PHEN] = 2:1) leads to an increase in the luminescence intensity *I*_614_ by more than one order of magnitude (by 13 times).

The intensity of luminescence *I*_614_ in solutions of complexes strongly depends on the comonomer’s nature. Table 3 presents the corresponding data for VPA copolymers with 4-AM, MAG, and AA of close compositions (14, 12, and 14 mol.% of copolymer units). As can be seen, the highest luminescence intensity was found for the VPA-4-AM copolymer. Apparently, this is related to the influence of the macromolecular environment of the phosphonate fragments on its ability to bind Eu^3+^, TTA, and PHEN, affecting, for example, the possibility of forming H-bonds between the units of neutral fragments and the P=O group in the copolymer, which can lead to a change in the binding constants of both Eu^3+^ ions and neutral ligands.

To summarize, the synthesized copolymers form complexes with Eu^3+^ that demonstrate the intense red luminescence.

## 4. Conclusions

Copolymers of vinylphosphonic acid (VPA) with 2-deoxy-2-methacrylamido-D-glucose and 4-acryloylmorpholine were synthesized for the first time; copolymers of VPA with acrylamide were also prepared. The products contained from 6 to 97 mol.% of VPA, and the molecular masses of the synthesized copolymers varied from 5 × 10^3^ to 310 × 10^3^. The reactivity ratios of vinylphosphonic acid and 2-deoxy-2-methacrylamido-D-glucose in copolymerization were determined, and it was found that vinylphosphonic acid was less active in these copolymerization processes than other comonomers. The resulting copolymers contained lower amounts of VPA units than the initial comonomer mixture in all cases. An increase in the VPA concentration in the initial mixture led to a decrease in the copolymer yield and molecular masses. Copolymerization in aqueous solutions yielded products with higher molecular masses than copolymerization in organic solvents (2,2-dimethylformamide or methanol).

It was demonstrated that the synthesized copolymers form complexes with Eu^3+^ ions in the presence of thenoyltrifluoroacetone and phenanthroline, while the intensity of the luminescence of these complexes in solutions in the long-wavelength region depends on the comonomer nature (2-deoxy-2-methacrylamido-D-glucose, 4-acryloylmorpholine, or acrylamide).

## Figures and Tables

**Table 1:** Synthesis conditions and characteristics of VPA copolymers (M_1_).

N	Conditions of Copolymerization ^a^						Characteristics of Copolymers		
	[M_2_]	[M_1_]:[M_2_], mol.%	[M_1_ + M_2_], wt.%	Solvent	I ^b^	[I], wt.% of [M_1_ + M_2_]	Yield, %	[M_1_], mol.%	M_SD_ × 10^−3^
1 ^c^	–	100:0	80	H_2_O	AMP	1	54	100	30
2	MAG	25:75	10	H_2_O	AMP	2	86	6	117
3	MAG	50:50	10	DMFA	AIBN	2	71	12	20
4	MAG	90:10	10	H_2_O	AMP	2	29	53	5
5	4-AM	25:75	20	Methanol	AIBN	2	76	13	77
6	4-AM	25:75	20	H_2_O	AMP	2	82	14	310
7	4-AM	50:50	20	H_2_O	AMP	1	38	56	33
8	4-AM	90:10	20	H_2_O	AMP	1	27	97	10
9	AA	25:75	20	Methanol	AIBN	2	77	13	40
10	AA	25:75	20	DMFA	AIBN	2	93	28	25
11	AA	25:75	20	H_2_O	AMP	2	74	14	240
12	AA	50:50	20	H_2_O	AMP	1	56	31	70
13	AA	90:10	20	H_2_O	AMP	1	11	63	7

^a^ 24 h, 60 °C, ^b^ I—initiator, ^c^ 80 °C.

**Table 2:** The *I*_614_ values for complexes of Eu^3+^ with 4-AM-VPA, TTA, and PHEN, in aqueous solutions.

Solution	I614 (U = 400)
Eu^3+^/4-AM-VPA copolymer	n/d
Eu^3+^/TTA	1
Eu^3+^/PHEN	<1
Eu^3+^/4-AM-VPA copolymer/TTA	186
Eu^3+^/4-AM-VPA copolymer/PHEN	72
Eu^3+^/4-AM-VPA copolymer/TTA/PHEN	2434

**Table 3:** The *I*_614_ values for complexes of Eu^3+^ with VPA copolymers in aqueous solutions in the presence of sensitizers.

N (Table 1)	M_2_	*I*_614_ Copolymer/TTA	*I*_614_ Copolymer/TTA/PHEN	*I*_TTA+PHEN_/*I*_TTA_
5	4-AM	92	1272	14
3	MAG	18	944	52
11	AA	3	204	68
